# A systematic review of the quality of conduct and reporting of survival analyses of tuberculosis outcomes in Africa

**DOI:** 10.1186/s12874-021-01280-3

**Published:** 2021-04-27

**Authors:** Moses M. Ngari, Susanne Schmitz, Christopher Maronga, Lazarus K. Mramba, Michel Vaillant

**Affiliations:** 1 KEMRI/Wellcome Trust Research Programme, P.O Box 230, Kilifi, 80108 Kenya; 2 The Childhood Acute Illness & Nutrition Network (CHAIN), Nairobi, Kenya; 3 Competence Center for Methodology and Statistics, Department of Population Health, Luxembourg Institute of Health, Strassen, Luxembourg; 4 Department of Biostatistics and Data Science, University of Kansas Medical Center, Kansas, USA

**Keywords:** Survival analysis, Time-to-event, Tuberculosis, Systematic review, Africa

## Abstract

**Background:**

Survival analyses methods (SAMs) are central to analysing time-to-event outcomes. Appropriate application and reporting of such methods are important to ensure correct interpretation of the data. In this study, we systematically review the application and reporting of SAMs in studies of tuberculosis (TB) patients in Africa. It is the first review to assess the application and reporting of SAMs in this context.

**Methods:**

Systematic review of studies involving TB patients from Africa published between January 2010 and April 2020 in English language. Studies were eligible if they reported use of SAMs. Application and reporting of SAMs were evaluated based on seven author-defined criteria.

**Results:**

Seventy-six studies were included with patient numbers ranging from 56 to 182,890. Forty-three (57%) studies involved a statistician/epidemiologist. The number of published papers per year applying SAMs increased from two in 2010 to 18 in 2019 (*P* = 0.004). Sample size estimation was not reported by 67 (88%) studies. A total of 22 (29%) studies did not report summary follow-up time. The survival function was commonly presented using Kaplan-Meier survival curves (*n* = 51, (67%) studies) and group comparisons were performed using log-rank tests (*n* = 44, (58%) studies). Sixty seven (91%), 3 (4.1%) and 4 (5.4%) studies reported Cox proportional hazard, competing risk and parametric survival regression models, respectively. A total of 37 (49%) studies had hierarchical clustering, of which 28 (76%) did not adjust for the clustering in the analysis. Reporting was adequate among 4.0, 1.3 and 6.6% studies for sample size estimation, plotting of survival curves and test of survival regression underlying assumptions, respectively. Forty-five (59%), 52 (68%) and 73 (96%) studies adequately reported comparison of survival curves, follow-up time and measures of effect, respectively.

**Conclusion:**

The quality of reporting survival analyses remains inadequate despite its increasing application. Because similar reporting deficiencies may be common in other diseases in low- and middle-income countries, reporting guidelines, additional training, and more capacity building are needed along with more vigilance by reviewers and journal editors.

**Supplementary Information:**

The online version contains supplementary material available at 10.1186/s12874-021-01280-3.

## Background

Application of survival analyses, in this article referred to as `*Survival analyses methods (SAMs)*’, have rapidly increased especially in oncology over the years [1]. They are used to analyze time-to-event outcomes and entail estimating; a) the probability of the outcome (event) of interest, b) the time the event occurs or c) exploring associations of time-to-event outcome with some independent predictors [2]. Therefore, SAMs usually provide more valuable information about how the probability of the event of interest changes with time compared to other standard statistical methods analyzing binary outcomes [2].

The probability of being event free at time *t*, usually denoted as *survival function* is commonly plotted using the Kaplan-Meier (KM) curve [2] while the probability of experiencing the event of interest; *the cumulative event function* is presented graphically using the Nelson-Aalen curve [3]. A life table is used to estimate and present survival time, but can only approximate the survival function within fixed intervals and is thus rarely used in survival analysis [4]. Log-rank tests are commonly used to compare the survival function between two or more groups [2].

The Cox Proportional Hazard (CPH) regression method, a semi-parametric model, is one of the most frequently used methods in survival regression analysis [5, 6]. The CPH model assumes the hazards are proportional over time (i.e. the hazard ratios are constant over time) [7]. Parametric proportional hazard models are similar but assume a specific statistical distribution for the hazard calculation and are considered more efficient because they estimate the baseline hazard rate [6, 8]. Additionally, SAMs have to take into account the non-informative censoring assumption (i.e. censoring time is statistically independent of their failure time) [2]. There are other broader considerations that are not SAMs specific and affect other applications of statistics such as appropriate assumptions when estimating sample size, lack of independence in presence of clustering or recurring events [2, 8–15].

Inappropriate conduct and low quality of reporting SAMs have been identified previously and may lead to incorrect conclusions [1, 16–18]. Previous published reviews of SAMs in medical research have found the quality of reporting SAMs inadequate [1, 16, 17, 19, 20]. The reviews included 764 studies (566 in oncology, 97 in cardiology, 73 in internal medicine, 14 in nephrology and 14 in acute lymphoblastic leukemia) conducted between 1991 and 2017. These reviews included only studies of non-communicable diseases predominately conducted in high income countries. All reviews identified significant deficiencies in reporting SAMs including non-reporting of sample size estimation and testing of the PH assumption in the CPH regression models. In addition, there have been reports of inadequate and incomplete reporting of randomized trials and studies on infectious diseases without statisticians/epidemiologists in Africa [21, 22].

Tuberculosis (TB) is an infectious disease that requires treatment for at least six months. It is one of the leading causes of deaths from a single infectious agent globally and usually shows worse outcomes when it occurs among HIV infected patients [23]. Globally, the highest burden of TB is from Sub-Saharan Africa [24]. This article provides the first systematic review of the quality of reporting SAMs in studies of TB patients in Africa. In this study we aim to review the application and reporting of SAMs in studies of TB patients in Africa published from January 2010 to April 2020 in English.

## Methods

### Study design

We conducted a systematic review of studies from Africa that included TB patients and reported SAMs. TB end of treatment outcomes defined by World Health organization (WHO) formed the basis for the time-to-event analyses in this review (cured, completed treatment, failed treatment, died, defaulted, transferred out and successful treatment) [25]. The study followed the Preferred Reporting Items for Systematic Reviews and Meta-Analyses (PRISMA) guidelines [26].

### Search strategy

A systematic search for eligible studies in MEDLINE via PubMed and EMBASE database was conducted in May 2020. The exact search terms are available in Additional file 1: Box 1.

### Selection criteria

Published papers were eligible for inclusion if they met the following criteria: i) the study population consisted of patients in Africa with TB (co-morbidity with other common infection like HIV was allowed); ii) follow-up data were available (i.e., cohort studies or randomized clinical trials); iii) SAM analysis methods were used; iv) the study was published between January 2010 and April 2020; v) the study was published in English language. Including papers published in the last ten years was deemed reasonable to capture recent trends in the application and reporting SAMs. We supplemented the search by reviewing references in the final list of articles that met eligibility criteria. Studies conducted in Africa but including sites outside Africa were excluded, however, where separate and complete analyses were conducted for each site, results from the African sites were included. We also excluded conference articles with abstracts only, protocols, methodology papers, systematic reviews and meta-analyses.

### Screening of studies

The references from both databases were exported to Endnote X8 [27] where duplicates were removed. The remaining studies were exported into a screening software, Rayyan web app [28]. Study selection based on inclusion and exclusion criteria was conducted in a two-stage screening process: two assessors (MMN and CM) screened each reference first based on title and abstract and second based on the full text. All disagreements were resolved through discussion by the two assessors.

### Data extraction

We extracted data from the included studies in a data extraction template (Additional file 1: Appendix 1) designed in REDCap database [29]. The template was finalized following a piloting phase ensuring its suitability. Two authors (MMN and CM) independently performed the data extraction of each included reference; disagreements were resolved through discussion. In studies that performed more than one survival analysis, the main analysis was included. The following details were extracted: year of publication, publication journal, study design, involvement of a statistician/epidemiologist, collaboration with authors outside Africa, TB treatment outcome, number of study participants, reporting of follow-up time, graphic presentation of time-to-event, method used for group survival comparison (where applicable), type of survival regression models, method of testing underlying assumptions of any used regression models, statistical software used, reporting of sample size calculations, reporting exposure variables missing data, testing of interactions in regression models, reporting of lost-to-follow-up, censoring description and inclusion of multiple study sites/clusters. Information about involvement of a statistician/epidemiologist was extracted from the authors’ affiliation, acknowledgement section or authors’ information at the end of the manuscript and covered broad subject of statistics, biostatistics or epidemiology. Censoring description was assessed by checking studies that reported any mention of censoring, type of censoring, mention of non-informative censoring assumption and any method used when the non-informative censoring was violated or not assumed. Items covering broader statistical consideration like sample size estimation were included to help unravel the bigger methodological aspect, for example, a study with inadequate statistical power would yield non-conclusive results despite the SAMs used.

### Evaluation of quality of reporting survival analyses

The quality of reporting survival analyses was assessed using seven author-defined criteria (Table 1). Items included in the seven criteria were based on key elements of survival analyses identified by Altman et.al [2, 6, 30, 31] and previous reviews [1, 16, 17] which were assessed by the authors, piloted and final items agreed upon. Through their experience, the authors, grouped the final items selected into the seven thematic survival analyses areas (the seven criteria). In brief, key survival analysis concepts and items previously evaluated were enumerated and organized into two domains: a) issues in design phase and b) statistical analysis phase. In design phase, sample size estimation and planned follow-up time were identified. Items identified during statistical analysis phase were grouped into five categories as presented in Table 1. Since this is a review of TB end-time outcomes, reporting consideration of recurring time-to-event in analysis was excluded.

**Table 1:** Criteria for evaluating quality of reporting SMAs

Criteria	Items assessed	Quality of reporting		
		Adequate	Inadequate	Not reported
Estimation of sample size	Statistical power; hypothesised effect estimate; effect size; alpha level; prevalence of exposure and probability of the expected outcome	All of these items reported for prospective studies. For retrospective studies, post hoc power estimation or detectable difference	At least one item was not reported	No sample size estimation information provided
Follow-up time	Start and exit dates and aggregate follow-up time (median/person-time)	Reported all these items	At least one item was not reported	None of the items was reported
Survival curves	Number of patients at risk at the bottom of the graph; markings to indicate when participants were censored; axes were clearly labelled and used different colors/type of lines to distinguish curves	Reported all these items	At least one item was not reported	No survival curve was plotted
Comparison of survival curves	Methods for group comparisons and their test results (p-values)	Reported all these items	At least one item was not reported	No comparison was done
Reporting measures of effect in SAMs	Measures of effect and uncertainty among studies reporting regression analysis	Correct measure of effect and uncertainty reported	Incorrect measure of effect or no measure of uncertainty	No measure of effect and uncertainty reported
Test of survival regression models underlying assumptions	Survival regression models used; statistical method used to test underlying assumptions and test results	Reported all these items	At least one of the items not reported	All the items not reported
Analysis of hierarchical clustering	Presence of clustering; methods of investigating heterogeneity and correct method for adjustment	Test of investigating heterogeneity and correct method for adjustment where there was evidence of heterogeneity reported	At least one of the items not reported	No consideration for clustering

### Statistical analysis

Frequency of studies reporting the seven evaluation criteria are reported with their respective percentage. We assessed the trend of the number of papers published across the years of publication from 2010 to 2019 using a Wilcoxon-type test for trend [32]. In a sub-analysis, we explored association of journal, year of publication and involvement of a statistician/epidemiologist with the quality of reporting (not reported, inadequate and adequately reported) using chi-square test/fisher’s exact test. However, the results of the sub-analysis are only indication of possible associations as no power analysis was performed during study design. STATA/IC (version 15.1; StataCorp, College Station, TX, USA) was used to perform statistical analysis.

## Results

### Search results

**Fig. 1:**
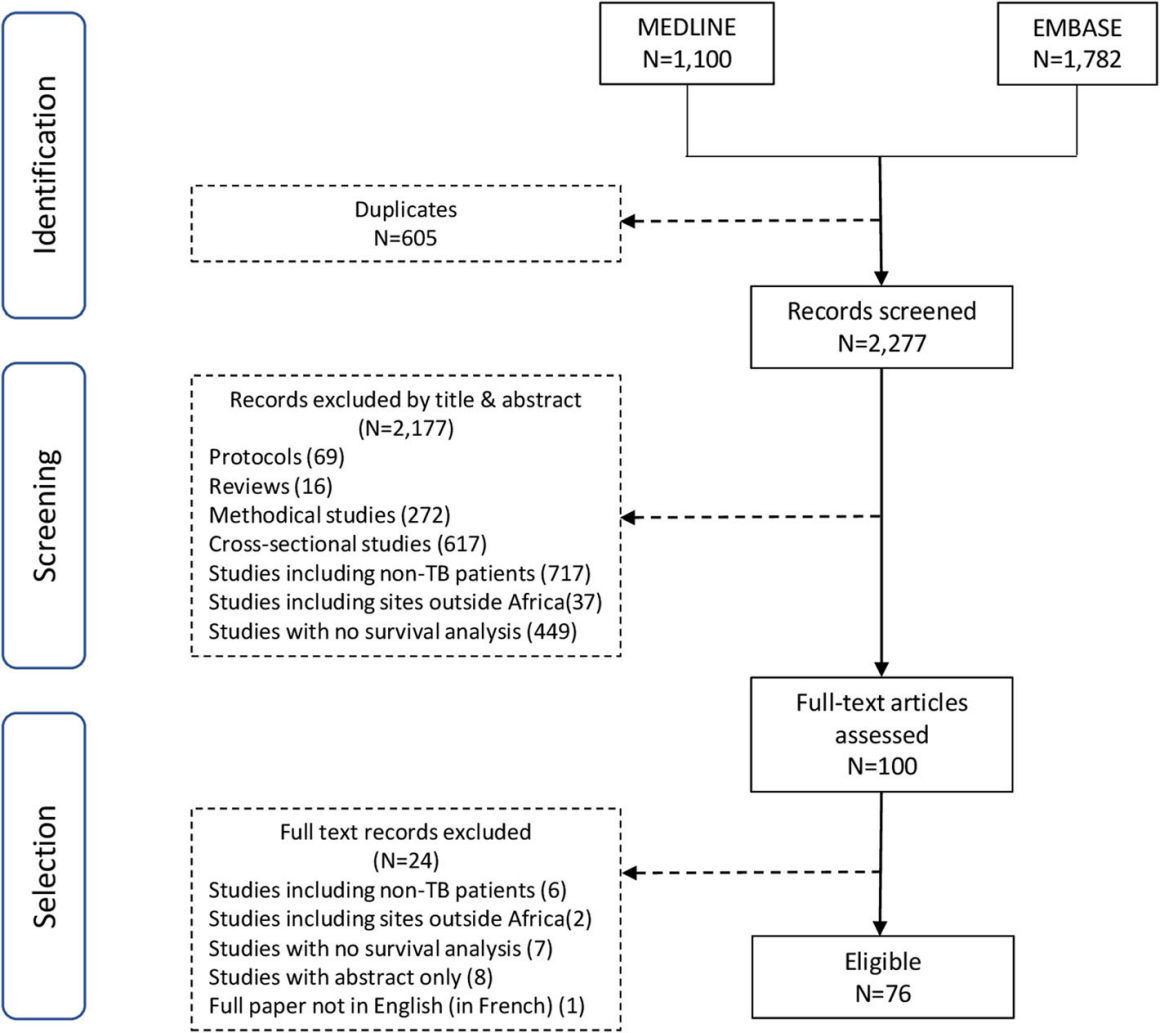
Study flow diagram showing how studies were selected

The search yielded 1100 studies from MEDLINE (PubMed) and 1782 from EMBASE (Fig. 1). Six hundred and five duplicates were removed. We excluded 2177 studies after screening titles and abstracts. We reviewed the full text for 100 studies of which we excluded 24. Therefore, 76 studies were eligible for inclusion in the analysis. The full list of the 76 studies included is provided in the Additional file 1: Table S1.

### Characteristics of included studies

**Fig. 2:**
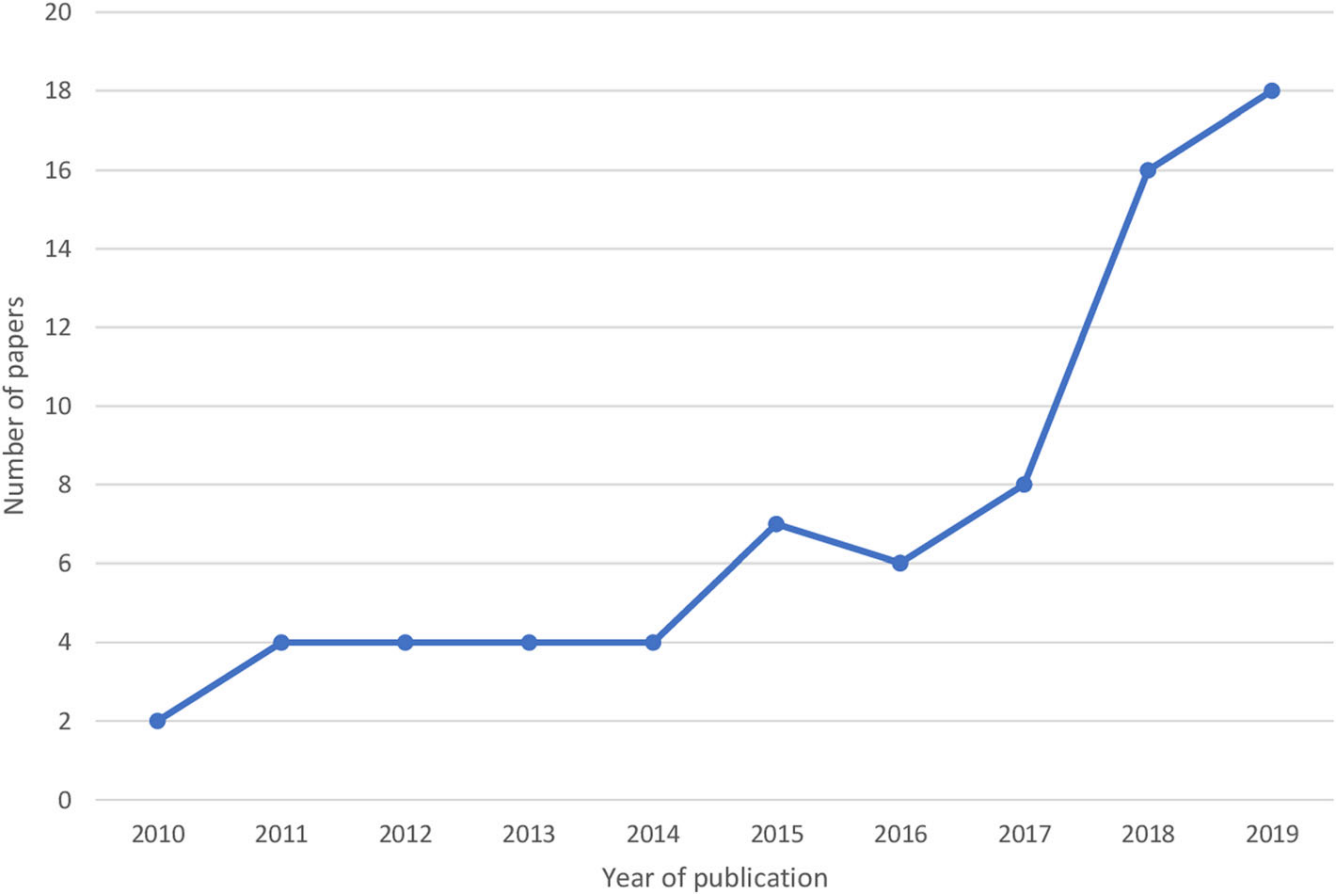
Trend of the annual number of papers using SAMs from 2010 to 2019. Trend p-value = 0.004

Characteristics of included studies are summarized in Table 2. Of the 76 studies, only one (1.3%) was a randomized trial, 54 (71%) were retrospective cohorts and 21 (28%) were prospective cohorts. Different time-to-event outcomes were evaluated with time to death (*n* = 72, (95%)) being the most common. The size of the studies ranged from 56 to 182,890 participants. Forty-three (57%) studies involved a statistician/epidemiologist in design or analysis. Collaborators from developed countries were included in 55 (72%) studies. STATA was the most commonly used software for data analysis in 40 (53%) studies, followed by SPSS (20%), SAS (12%) and R statistical programming (*n* = 7, (9.2%)). Five (6.6%) studies did not report the statistical software used [33–37]. Articles were most frequently published in PLOS One, International Journal of Tuberculosis and Lung Diseases (IJTLD) and BMC Infectious Disease; accounting for 53% of studies (Table 2). The number of published papers per year reporting SAMs increased from two in 2010 to 18 in 2019 (*P* = 0.004) Fig. 2.

**Table 2:** Characteristics of studies included in the review

	All the studies (***n*** = 76) n (%)
Study design	
Retrospective cohort	54 (71)
Prospective cohort	21 (28)
Randomized controlled trial	1 (1.3)
Type of study time-to-event outcome^a^	
Cured	9 (12)
Treatment complete	27 (36)
Treatment failure	16 (21)
Death	72 (95)
Default	37 (49)
Transfer out	5 (6.6)
Study size	
Median [IQR]	492 [286–1330]
Minimum to Maximum	56–182,890
Survival analyses objective	
Curve estimation	2 (2.6)
Survival regression analyses	16 (21)
Both	58 (76)
Involvement of statistician/epidemiologist	
Yes	43 (57)
Not reported	54 (71)
Authors affiliation^b^	
Country of focus only	21 (28)
Country of focus plus other African country	5 (6.6)
Country of focus plus developed country	55 (72)
Publication Journal	
PLOS One	22 (29)
International Journal of Tuberculosis and Lung Diseases (IJTLD)	9 (12)
BMC Infectious Disease	9 (12)
BMC Public Health	4 (5.3)
Clinical Infectious Disease	3 (3.9)
Others^c^	29 (38)
Statistical software used	
SPSS	15 (20)
SAS	9 (12)
STATA	40 (53)
R	7 (9.2)
Not reported	5 (6.6)

IQR interquartile range, ^a^Studies evaluated more than one time-to-event outcome, ^b^some studies had authors with African and developed countries affiliation and therefore the percentage > 100%, ^c^AIDS-1, AIDS Respiratory Therapy-1, BMJ Thorax-1, American Journal of Tropical Medicine and hygiene-1, Annals of Epidemiology-1, EclinicalMedicine-1, Infectious diseases-1, Infection-1, Infectious Diseases of Poverty-2, International Journal of Infectious Diseases-2, International Journal of Mycobacteriology-2, International Journal of Pharmaceutical and Clinical Research-1, Journal of Acquired Immune Deficiency Syndromes-1, Lancet-1, Lancet Respiratory Medicine-1, PLOS Medicine-2, Pan African Medical Journal-1, The Pediatric Infectious Disease Journal −3, The Journal of Pediatrics-1, The Journal of Infectious Diseases-1, Tropical Medicine and International Health-2, Tropical Medicine and Health-1

### Evaluation of reporting

#### Estimation of sample size

Very few (*n* = 9, (12%)) of the studies reported sample size estimation (Table 3), of which 3 (4.0%) did so adequately and 6 (7.9%) inadequately (Table 5).

**Table 3:** Reporting of follow-up time, plotting of survival curves and survival regression analyses

	Number of articles n (%)
Sample size estimation reported	
Yes	9 (12)
Not reported	67 (88)
Reporting of follow-up time	
Median time	28 (37)
Person time	26 (34)
Not reported	22 (29)
Survival curves	
Kaplan-Meier	51 (67)
Nelson-Aalen	14 (18)
Not reported	11 (15)
Comparison of survival curves	
Log-rank test	44 (58)
Weighted log-rank test (Wilcoxon-Breslow-Gehan)	1 (1.3)
Not reported	31 (41)
Survival Regression models (*N* = 74)	
Cox proportional hazard	67 (91)
Competing risk analysis	3 (4.1)
Parametric proportional hazard	2 (2.7)
Parametric accelerated failure time	2 (2.7)
Reported regression models assumptions tested^a^	
Cox PH (*N* = 67)	
Visual (graphical log-log plots)	11 (16)
Schoenfeld residuals test	21 (31)
Not reported	35 (52)
Competing risk analysis (N = 3)	
Schofield residuals test	1 (33)
Not reported	2 (67)
Parametric Methods (N = 4)	
Information theory (LL, AIC, BIC)	4 (100)

^a^These are reported methods used to test the underlying assumptions in the statistical methods section and not the actual number of studies that reported the test results, PH-Proportional Hazard, LL-likelihood values, AIC-Akaike Information Criteria, BIC-Bayesian Information Criteria

#### Follow-up time

More than two thirds (*n* = 54, (71%)) of the studies reported follow-up time (Table 3): 52 (68%) adequately and 2 (2.6%) inadequately (Table 5).

#### Survival curves

**Fig. 3:**
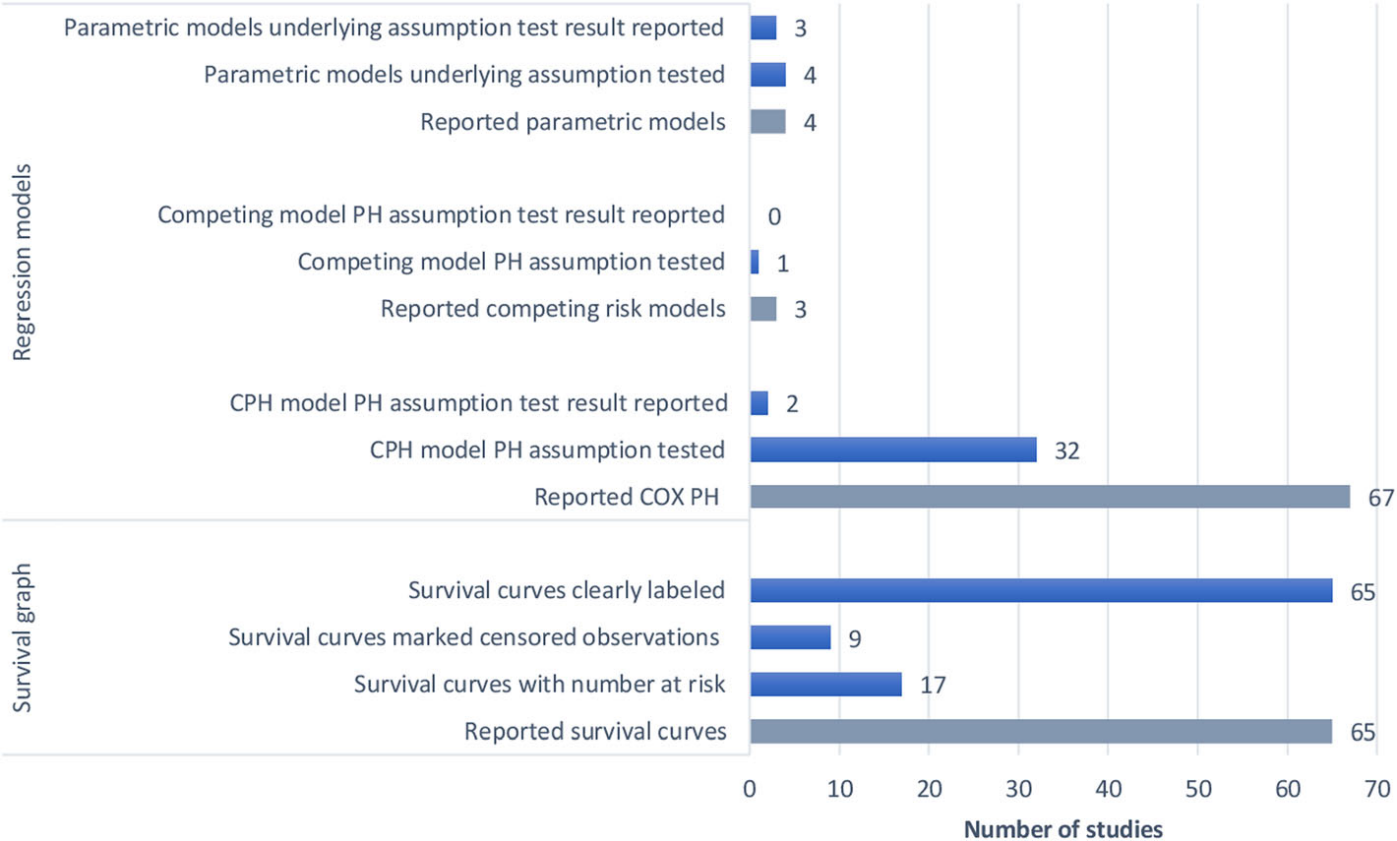
Bar graph of the survival plots and type of regression models reported. CPH, Cox Proportional Hazard; PH, Proportional Hazard, bar with grey color represent the total number of studies included in each subgroup

Survival curves were reported by 65 (86%) of the studies: Kaplan-Meier graphs were shown by 51 (67%) and Nelson-Aalen cumulative curves by 14 (18%) studies (Table 3). However, of the 14 studies reporting Nelson-Aalen cumulative curves, 9/14 (64%) were labelled as Kaplan-Meier [38–46]. Among the 65 studies reporting survival curves, 17/65 (26%) reported the number of patients at risk at each time point, 9/65 (14%) marked the survival time for the censored observations and all the 65 (100%) clearly labelled lines for different curves (Fig. 3). The reporting of survival curves was adequate in 1(1.3%) and inadequate in 64 (84%) of the studies (Table 5).

#### Comparison of survival curves

The survival function estimator curves were compared between groups in 45 (59%) studies either by using log-rank test (*n* = 44, (58%)) or weighted log-rank test (Wilcoxon-Breslow-Gehan) (*n* = 1, (1.3%)) and all the 45 studies reported the test *p*-values (Table 3). All the 45 studies adequately compared the survival distributions (Table 5).

#### Reporting measures of effect

Seventy-four (97%) studies performed survival regression analysis: 67 (91%) using CPH model, 3 (4.1%) competing risk analysis, and 4 (5.4%) parametric models. Two of the studies applying parametric proportional hazard models used Gompertz and Weibull probability distributions [47, 48], while 2 studies fitted an accelerated failure time parametric models, both using Weibull probability distributions [49, 50].

The two studies reporting parametric accelerated failure time [49, 50] and 69 studies performing Cox (67 studies) and parametric (2 studies) proportional hazard models reported time ratios (TR) and hazard ratios (HR) as the measure of effect respectively. Two of the three studies that performed competing risk analysis reported sub-distribution hazard ratios (SHR) [45, 51] while the other study reported HR [52]. All 74 studies reported 95% confidence intervals as measure of effect uncertainty (Table 3). The reporting of measures of effect was adequate among 73 (96%) and inadequate in 1 (1.3%) study (Table 5).

#### Test of regression models underlying assumptions

Among 67 studies that performed CPH regression analysis, 32/67 (48%) mentioned testing of the PH assumption (in the statistical methods section), however, only 2/67 (3.0%) reported the PH assumption test result [42, 53]. Where the PH assumption was violated, some studies excluded individual predictors violating the assumption [54] or reported odds ratio rather than hazard ratios [55] or censored the analysis at 28 days for a study with follow-up of 12 weeks to meet the PH assumption [56]. Only one study [51] among the 3 that performed competing risk analysis mentioned testing the underlying PH assumption but did not report the test results. The two studies that used parametric PH methods tested the PH assumption using the Schoenfeld residual test and reported the results [47, 48]. All four studies (100%) that used parametric regression models reported testing the most fitting probability distribution using the maximum likelihood (LL), minimum Akaike Information Criteria (AIC) or Bayesian Information Criteria (BIC) and visual assessment of the Cox-Snell residual plots [47–50]. Three of the four studies (75%) reported the values of the LL, AIC and BIC for the different distributions assessed (Weibull, Exponential, Gompertz, log Logistic and Log-normal) and also plotted the Cox-Snell residual plots for all the distributions tested [47, 48, 50] (Table 3 and Fig. 3). The reporting of test of survival regression models’ assumption was adequate and inadequate among 5 (6.6%) and 32 (42%) studies respectively (Table 5).

#### Analysis of hierarchical clustering

Thirty seven (49%) studies had hierarchical clustering, some recruiting patients from multiple African countries [37, 57] or from one country but across widely dispersed hospitals with possible varying TB incidence. None of the 37 studies reported whether they assessed evidence of heterogeneity across the clusters. However, 9/37 (24%) of these studies reported consideration of clustering in the regression analysis (Table 4). All the 9 studies adequately controlled for the clustering in the analysis (Table 5).

**Table 4:** Reporting of important other analytic considerations

	Number of articles n (%)
Analysis of studies with hierarchical clustering (*N* = 37)	
No clustering consideration	28 (76)
Variance corrected method	4 (11)
Frailty models	3 (8.1)
Multilevel regression	2 (5.4)
Censoring description	
Yes	50 (66)
No	26 (34)
Test of effect modification/interaction (N = 4)	
Likelihood ratio test	2 (50)
Chi-square test of homogeneity	1 (25)
Not reported	1 (25)
Handling of missing exposure data	
Single imputation	2 (2.6)
Multiple imputation	3 (4.0)
A missing category included	1 (1.3)
Not reported	70 (92)

**Table 5:** Overall quality of reporting SAMs

Criteria	Quality of reporting		
	Not reported	Inadequate	Adequate
Sample size estimation	67 (88)	6 (7.9)	3 (4.0)
Follow-up time	22 (29)	2 (2.6)	52 (68)
Plotting of survival curves	11 (14)	64 (84)	1 (1.3)
Comparison of survival curves	31 (41)	0	45 (59)
Test of regression underlying assumptions	39 (51)	32 (42)	5 (6.6)
Reporting measures of effect	2 (2.6)	1 (1.3)	73 (96)
Analysis of hierarchical clustering (N = 37)	28 (76)	0	9 (24)

#### Description of other statistical methods

Fifty (66%) studies reported censoring description. The majority (*n* = 46, (92%)) right censored participants following study completion, death, lost-to-follow-up or transfer out. Only one study reported investigating the non-informative censoring assumption by plotting observed survival times against values of the independent variables included in the regression models, and reported the assumption was not violated [35]. However, 4 (8%) studies reported considering the non-informative censoring assumption and adjusted the analysis using competing risk models (3 studies) and inverse probability censoring weighting (1 study) [58]. Four (5.2%) studies reported testing for some effect modification or interactions in the regression model [59–62] and provided stratified analyses where there was evidence of effect modification. A total of 70 (92%) of the studies did not report the proportion of missing exposure variables data or how the missing data were handled in the analysis (Table 4).

#### Overall evaluation

Adequate reporting was high for reporting measures of effect and their uncertainty (*n* = 73, (96%)), follow-up time (*n* = 52, (68%)) and comparison of survival curves (*n* = 45, (59%)). However, adequate reporting was very low for sample size estimation (*n* = 3, (4.0%), plotting of survival curves (*n* = 1, (1.3%)) and testing of underlying regression models assumptions (n = 5, (6.6%)). Approximately one quarter (24%) of studies adequately reported consideration of clustering in the regression models (Table 5).

In the sub-analyses, we found no evidence of journal, year of publication and involvement of a statistician/epidemiologist association with the quality of reporting SAMs (all *P*-values > 0.05).

## Discussion

In this systematic review of studies spanning over ten years, we found fundamental deficiencies in the reporting of survival analyses and an increasing trend in papers reporting SAMs annually. Sample size estimation, plotting of survival curves and assessment of regression underlying assumptions were rarely adequately reported. These deficiencies may lead to bias in reported measures of effect estimates and inaccurate conclusions. These are not isolated findings, as previous studies focusing on the quality of reporting SAMs [1, 16, 17, 19, 20], observational studies [63, 64] and even clinical trials [22, 65, 66] reported similar inadequacy. However, our analysis showed adequate reporting of effect measures.

Unlike a previous review of studies published in cancer journals, follow-up time and comparison of survival curves were frequently reported adequately [16]. However, some authors did not correctly distinguish Kaplan-Meier and Nelson-Aalen cumulative curves. Although Kaplan-Meier curves were commonly reported, in practice Nelson-Aalen curves plotting cumulative proportions of patients who experience the event are more informative [67]. In two previous reviews [1, 16] and this review, log-rank test was frequently reported probably because of its simplicity [2]. However, its *p*-value may not provide much information about the probabilities of an event at different time points and therefore providing a measure of survival time in each group like median survival time would be more useful. A log-rank test is most appropriate when the PH assumption is met [68–70], an alternative is the weighted log-rank test which assigns weights proportional to the contribution of each failure time [68, 71–74] but was rarely used.

Just like previous reviews [1, 17, 19], CPH regression models were used in the majority studies. Although 48% of the studies did mention that they evaluated the PH assumption using either visual (graphical log-log plots) or residuals tests (Schoenfeld), only 3.0% reported the test results. In a review of 14 studies that used CPH models, none reported assessing the PH assumption [1] while another review of application of SAMs in clinical trials found only 2/28 (7.1%) reported assessing the PH assumptions [19]. Similarly, amongst 112 Chinese Oncology studies that used CPH models, none reported assessing the PH assumption [20]. Only four studies used parametric methods and reported assessment of the underlying assumptions. All the four studies involved a statistician/epidemiologist, a demonstration of the central role they play. When correctly specified, parametric models are more efficient and informative because they provide an estimate of baseline hazard ratio that can be used in predicting absolute risks [30, 75].

Our findings suggest many authors were not aware of the alternatives to use when PH assumption is violated and resulted to incorrect methods like excluding independent variables found to have violated the PH assumption [54]. When the PH assumption is violated for some continuous variables, creating binary or ordinal variables could be an option [30]. Alternatively, the variables could be included as time-varying predictors or time stratified analysis could be performed [30]. Parametric accelerated failure time models measure the effect of the covariate on a time scale rather than hazard scale and do not assume the PH assumption. They have been shown to be more robust in oncology and may be considered too [9, 76]. Restricted mean survival time (RMST) which reports the difference in RMST as a measure of effect at suitable follow-up time as been suggested as other alternative when PH assumption is violated [77]. A possible reason for many authors to not report test results of model assumptions may be journal’s restrictions in the number of tables/figures allowed. The three studies that extensively reported the AIC, LL and BIC test results and plotted the cox-Snell plots were published in journals that do not limit number of tables/figures [47–49]. However, in the sub-analysis we found no evidence of association between the journal of publication and any of the reporting criteria. We would recommend journals to encourage authors to report these test results in the supplementary appendix as an extension of the statistical methods.

In presence of competing events, the Kaplan-Meier function produces biased estimates. When the time-to-event of interest is treatment success, it is plausible to assume other treatment outcomes such as ‘death’, `lost-to-follow-up’ and `transfer out’ were informative censored and thus considered as competing events. Fine and Gray non-parametric test comparing the cumulative incidence functions without requirement of non-informative censoring could be used in such settings [10, 78–81]. However, application of this method was rare in this review and one of the studies using the method, incorrectly reported hazard ratios rather than sub-distribution hazard ratios [52]. Other methods like inverse probability censoring weighting and some proposed methods using predicted long-term vital status may yield more accurate measures of effect estimates [82, 83]. Violation of non-informative censoring assumption may result in biased measure of effect estimates and thus should be investigated and appropriate adjustment made in the analysis although this was rarely done in the papers reviewed [82, 83].

Only 4% studies adequately reported the estimation of sample size, which is a key ingredient in any study design and a factor in determining the power to yield valid results. In a systematic review of lymphoblastic leukemia literature, 4/14 (29%) studies reported estimation of the study size which is slightly higher than our finding [1]. Since 71% of the studies were retrospective cohorts in this review, its likely they analyzed all the available records, but in such settings authors should be encouraged to perform a priori sample size estimation [84].

More than three quarters of the studies with some form of clustering of participants did not consider the design aspect in the analysis. This may point to a major challenge in the analysis of such designs despite there being comprehensive statistical methods of investigating cluster heterogeneity and controlling for the extra level of variation [31, 85, 86]. Not accounting for clustering in the analysis, may yield biased and extreme results leading to a false conclusion [13]. However, it was encouraging to observe, all the statistical software reported have robust systems to handle survival analyses, investigate and perform adjustments for non-informative censoring and clustering. Reporting of sample size estimation and accounting for clustering in analysis are not SAMs specific issues but the low frequency of adequacy of their reporting in this review, raises the possibility of suboptimal practices across reporting of TB in general.

The Consolidated Standards of Reporting Trials (CONSORT) and Strengthening the Reporting of Observational Studies in Epidemiology (STROBE) guidelines were developed to harmonize and improve quality of reporting randomized control trials (RCTs) and observational studies respectively, however, their focus is not on specific statistical methods [87, 88]. Recommendations on how to report specific statistical topics like missing data imputation [89], Bayesian analysis [90], and logistic regression [91] have been developed. Apart from suggestions by two previous reviews of SAMs [16, 17], currently there is no recommended standard guidelines for reporting SAMs. From our findings, we propose some pragmatic recommendations (Table 6) for researchers, statisticians and journal editors and emphasize the need to develop harmonized guideline for reporting SAMs.

**Table 6:** Recommendation for reporting survival analyses methods

Section	Recommendation
Study design	• Define the study time-to-event outcome. • Report the sample size and sample estimation methods providing all the assumptions made in calculating sample size. • Report the planned fixed length of follow-up (days, months, years).
Statistical methods and results	• Report beginning and end dates of each event under observation. • Report the total time under observation using standard epidemiological units like person-years and median time. Where the aim is to compare groups of participants, in additional to total time observed, report total time and median time stratified by the groups. • Report number of participants lost-to-follow-up, how censoring was done and if non-informative censoring assumption was evaluated. • Report total number of time-to-event outcome events observed, and events per groups. • Provide the survival probabilities at specific follow-up time points (outcome free probabilities where the outcome is not death), median survival time and 95% confidence interval is preferred for comparison with other studies. This should be provided for each group as well when the objective is to compare groups. • Report the method used to estimate the survival probabilities and plot the survival curves using appropriate graphs like Kaplan-Meier or Nelson-aalen cumulative curve stratified by groups when necessary. Include the following information in the survival curves: number of participants at risk at each specified timepoint, indicate when participants were censored, use different colors/type of lines to distinguish group curves and clearly label the x-axis as time under observation and y-axis appropriately. • When testing hypothesis of differences in survival probabilities between/among groups, report the method used, the test results and a P-value. • When survival regression is performed, report the methods used to test underlying assumptions (test for Proportion Hazard assumption for Cox regression and test of used probability distribution for parametric methods) and the test result. • Report the measure of effect (e.g Hazard ratios, sub-distribution hazard ratios, time ratios), their measure of uncertainty (e.g 95% confidence intervals, standard errors) and *P*-values from the regression model. • Like other statistical regression modeling, report all the covariates assessed, method of selecting features to be included in the multivariable survival regression model, methods used to assess the multivariable regression goodness of fit, proportion of missing data in the outcome and covariates assessed plus how missing data were handled, methods used to test for interaction and methods used to control for clustering in multilevel studies.

Excluding non-English papers was one of the study limitations. However, looking at the countries where the studies were conducted, suggests the Francophone and other non-English speaking countries (like Ethiopia and Mozambique) were not excluded but could be underrepresented. The reporting of SAMs may be influenced by many factors like involvement of statistician/epidemiologist, but it was challenging ascertaining involvement and level of skills of the statistician/epidemiologist and the likely lack of power to perform such analysis. We thus explored the effect of such factors in sub-analysis.

## Conclusion

The quality of reported survival analyses in studies of TB in Africa is inadequate despite the increasing number of annual publications on the topic. Our findings suggest sample size estimation, testing of underlying survival regression models and visual display of the survival function were rarely adequately reported. Some of these deficiencies may lead to incorrect results and conclusion. Because similar reporting deficiencies may be common in other diseases in low- and middle-income countries, reporting guidelines, additional training and more capacity building are needed along with more vigilance by reviewers and journal editors.

## Supplementary Information

**Additional file 1.**

## Data Availability

The datasets used and analyzed in this current study are available from the corresponding author on reasonable request.

## Abbreviations

AIC: Akaike Information Criteria
ALL: Acute lymphoblastic leukemia
AUCs: Area under receiver operating characteristic curve
BIC: Bayesian Information Criteria
CPH: Cox Proportional Hazard
HIV: human immunodeficiency viruses
HR: Hazard ratios
IJTLD: International Journal of Tuberculosis and Lung Diseases
KM: Kaplan-Meier
LL: Likelihood
PH: Proportional Hazard
REDCap: Research electronic data capture
RMST: Restricted mean survival time
SAM: Survival analyses methods
SHR: Subhazard ratios
TB: Tuberculosis
TR: Time ratios
WHO: World Health organization
