# Supplementary material for: A systematic review of the quality of conduct and reporting of survival analyses of tuberculosis outcomes in Africa
Source: BMC Med Res Methodol. 2021 Apr 27;21:89. doi: 10.1186/s12874-021-01280-3 (PMC8080365; doi:10.1186/s12874-021-01280-3)
Supplement: Supplementary file 1 — Additional file 1. [file 12874_2021_1280_MOESM1_ESM.docx]

**Additional File**

**A systematic review of the quality of conduct and reporting of survival analyses of tuberculosis outcomes in Africa.**

Moses M. Ngari, Susanne Schmitz, Christopher Maronga, Lazarus K. Mramba, Michel Vaillant

Table of Contents

[Box 1. **Search strategy** 3](#_Toc62754538)

[Appendix 1. **Data extraction template** 4](#_Toc62754539)

[Table S1. **List of all the 76 studies included.** 8](#_Toc62754540)

[**References** 14](#_Toc62754541)

# Box 1. **Search strategy**

| (((((((Tuberculosis [MH]) OR (Tuberculosis[TI])) OR (Tuberculoses[TI])) OR (“TB disease”[TI])) AND ((((((Observational Study[PT]) OR (Observational[TIAB])) OR (“Cohort Studies”[MH])) OR (Cohort[TIAB])) OR (“Long-term”[TIAB])) OR (“Follow-up”[TIAB))) AND (((((((((((((((Treatment Outcome[MH]) OR (“Treatment success"[TIAB])) OR (Outcome[TIAB])) OR (Outcomes[TIAB])) OR (Effectiveness[TIAB])) OR (Efficacy[TIAB])) OR (mortality[TIAB])) OR (death[TIAB])) OR (Surviv*[TIAB])) OR (cured[TIAB])) OR (completed[TIAB])) OR (Default[TIAB])) OR (“Treatment failure"[TIAB])) OR ("Lost to follow-up"[TIAB])) OR ("Transfer out"[TIAB]))) AND ((Africa [MH]) OR (Africa*[TW]))) AND (("2010/01/01"[Date - Publication] : "2020/04/30"[Date - Publication])). |
| --- |

# Appendix 1. **Data extraction template**

| **Basic information** | |
| --- | --- |
| Study reference number | __ __ |
| Year of publication (2010 to 2020) | *__ __ __ __* |
| Publication journal | *_________________________* |
| Which country was study done? | _________________________ |
| Type of the published study | 🞎 Prospective cohort  🞎 Retrospective cohort  🞎 Randomized Trial |
| Total sample size reported? | 🞎 Yes  🞎 No |
| Total sample size [If yes above] | *__ __ __ __ __* |
| Did the study involve time-to-event TB treatment outcome? | 🞎 Yes  🞎 No |
| If YES, select the TB treatment outcome | 🞎 Cured  🞎 Treatment complete  🞎 Treatment failure  🞎 Death  🞎 Default  🞎 Transfer out  🞎 Treatment success |
| Number of outcome events reported | 🞎 Yes  🞎 No |
| Was a statistician/epidemiologist involved in the study? | 🞎 Yes  🞎 No  🞎 Not reported |
| Type of research collaboration | 🞎 Authors from county of focus only  🞎 Authors from other Africa state  🞎 Authors from developed countries (USA/Europe/Canada)  🞎 Others _____________________ |
| Type of analysis | 🞎 Curve estimation  🞎 Regression analysis  🞎 Both |
| If curve estimation, was any other type of regression performed?  [if curve estimation] | 🞎 Yes  🞎 No |
| Name the other type of regression [if YES above] | 🞎 Logistic regression  🞎 Log-binomial regression  🞎 Poisson regression  🞎 Negative binomial regression  🞎 Linear regression  🞎 Others ___________________ |

| **Reporting of descriptive SAMs** | |
| --- | --- |
| Follow-up time reported? | 🞎 Yes  🞎 No |
| Method of calculating follow-up time (if YES above) | 🞎 Median time  🞎 Person-time  🞎 Others ___________ |
| Start and end dates of follow-up reported? | 🞎 Yes  🞎 No |
| Graphical presentation of time-to-event reported? | 🞎 Yes  🞎 No |
| Graphical method reported (If YES above) | 🞎 Kaplan-Meier estimator  🞎 Nelson-Aalen estimator  🞎 Life time estimator |
| Patients number at risk at each time point reported in the graph? | 🞎 Yes  🞎 No |
| Censored observation marked on survival graphs/explained? | 🞎 Yes  🞎 No |
| Different curves well labeled on the graphs? | 🞎 Yes  🞎 No |
| Methods distinguishing the different curves (If yes above) | 🞎 Different colors  🞎 Different line types |

| **Survival analysis inferential statistical analyses reported** | |
| --- | --- |
| Any comparison of survival curves? | 🞎 Yes  🞎 No |
| Method of comparison (if YES above) | 🞎 Log-rank test  🞎 Wilcoxon-Breslow-Gehan test  🞎 Tarone-Ware test  🞎 Peto-Peto-Prentice test  🞎 Wald test  🞎 Unknown/not reported |
| Any measure of effect reported, like HR, RR? | 🞎 Yes  🞎 No |
| Measures/range of uncertainty reported (for the measure of effect); CIs, SEs | 🞎 Yes  🞎 No |
| What measures of uncertainty was reported? (If YES above) | 🞎 Confidence intervals  🞎 Standard Errors |
| Methods used to generate measure of effect reported? | 🞎 Yes  🞎 No |
| Methods used to generate measure of effect (If YES above) | 🞎 Mantel-Haenszel/cox  🞎 Cox PH  🞎 Accelerated failure time  🞎 Parametric methods  🞎 Competing risk analysis |
| Specify the parametric model used (If Parametric methods reported) | 🞎 Exponential  🞎Weibull  🞎 Gompertz  🞎 Log-Logistic  🞎 Log-normal  🞎 Generalized Gamma (GG)  🞎 Spline approach |
| Testing of used model underlying assumptions reported? | 🞎 Yes  🞎 No |
| Statistical methods used to test underlying assumptions (If YES above) | 🞎 Visual (graphical log-log)  🞎 Residuals approach (Schofield/Cox-snell)  🞎 Information theory (AIC, BIC, likelihood ratios)  🞎 Others__________________ |
| Results of the underlying assumptions test reported? (as P-values or AIC/BIC values or graphs) | 🞎 Yes  🞎 No |
| Statistical method used supported by test above? | 🞎 Yes  🞎 No  🞎 No results provided |
| Where there was underlying assumptions violation, was an alternative method used? (If NO above) | 🞎 Yes  🞎 No |
| Was the alternative method statistically appropriate? | 🞎 Yes  🞎 No |
| Explain the alternative method | ____________________________ |
| For regression models, was predictors selection strategy reported? | 🞎 Yes  🞎 No |
| What was the regression model building strategy? | 🞎 None (fixed variables used)  🞎 Stepwise approach  🞎 Penalized models  🞎 Criterion-based  🞎 Others____________________ |
| Was any statistical software used reported? | 🞎 Yes  🞎 No |
| What statistical software was used (If YES above) | 🞎 SPSS  🞎 SAS  🞎 STATA  🞎 R  🞎 Others_____________________ |

| **Advanced inferential analyses consideration reported** | |
| --- | --- |
| Sample size calculation appropriate for time-to-event outcome (check the statistical power, alpha, probability of outcome events, effect size (where applicable)) | 🞎 Yes  🞎 No |
| What parameters were specified in sample size estimation? If Yes above | 🞎 Statistical power  🞎 Alpha  🞎 Effect size  🞎 Probability of outcome event  🞎 Proportion of assumed exposure |
| Was there hierarchical clustering in the study? (Check clustering or clustered randomized trial) | 🞎 Yes  🞎 No |
| Was heterogeneity investigated (If yes above) | 🞎 Yes  🞎 No |
| Was appropriate statistical method used to account for clustering in survival analysis? | 🞎 Yes  🞎 No |
| What method was used to account for the clustering? If YES above. | 🞎 Variance corrected models  🞎 Multilevel regression  🞎 Frailty models  🞎 Others_____________________ |
| Was censoring description reported? | 🞎 Yes  🞎 No |
| What methods was used to adjust for informative censoring? | 🞎 Competing risk models  🞎 Inverse probability weighting  🞎 Sensitivity analysis  🞎 Others_____________________ |
| Did the study involve recurrent events (multiple outcomes)? | 🞎 Yes  🞎 No |
| Was appropriate statistical method used to account for recurrent events in survival analysis? | 🞎 Yes  🞎 No |
| What method was used to account for recurrent events? | 🞎 Variance corrected models  🞎 Multilevel regression  🞎 Frailty models  🞎 Others______________________ |
| Was effect modification/interaction tested? | 🞎 Yes  🞎 No |
| Effect modification/interaction test performed | 🞎 Likelihood ratio test  🞎 Mantel-Haenszel  🞎 Others______________________ |
| Was number of lost-to-follow-up reported? | 🞎 Yes  🞎 No |
| What was the proportion (%) of LTFU reported? If yes above. | *__ __%* |
| Was proportion of missing exposure data reported? | 🞎 Yes  🞎 No |
| Method of handling missing data | 🞎 No missing data  🞎 Not reported/unknown  🞎 Single imputation  🞎 Multiple imputation  🞎 Sensitivity analysis  🞎 Others______________________ |
| Was multivariable regression goodness of fit tested? | 🞎 Yes  🞎 No |
| Goodness of fit method used [If Yes above] | 🞎 None/not reported  🞎 Hosmer and Lemeshow test  🞎 AUCs/C-index  🞎 Log-likelihood ratio  🞎 AIC  🞎 R^2^  🞎 Residual analyses  🞎 Others_____________________ |
| Was multivariable regression model validated? | 🞎 Yes  🞎No |
| Model validation method used? [If YES above] | 🞎 Resampling (boot strapping)  🞎 Internal validation  🞎 External validation  🞎 Others____________________ |

| Table S1. **List of all the 76 studies included.** | | | | | | | | | | | |
| --- | --- | --- | --- | --- | --- | --- | --- | --- | --- | --- | --- |
| **Number** | **Primary Author** | **Year of publication** | **Publication Journal** | **Country of study** | **N included** | **Study design** | **Statistician among authors’ affiliation** | **Type of research collaboration** | **Method of event rate comparison** | **Regression model** | **Reference** |
| 1 | Tola et.al | 2020 | PLoS ONE | Ethiopia | 3478 | Retrospective cohort | Yes | Authors from country of focus and Iran | Log-rank test | Cox Proportion hazard model | [1] |
| 2 | Shaweno et.al | 2020 | Tropical Medicine and Health | Ethiopia | 1341 | Retrospective cohort | Yes | Authors from country of focus | Log-rank test | Cox Proportion hazard model | [2] |
| 3 | Schwoebel et.al | 2020 | EClinicalMedicine | Benin, Burkina Faso, Burundi, Cameroon, Central African Republic, Cote d’Ivoire, Democratic Republic of Congo, Niger and Rwanda | 1006 | Prospective cohort | Yes | Authors from country of focus and Europe | Not reported | Cox Proportion hazard model | [3] |
| 4 | Musaazi et.al | 2019 | International journal tuberculosis lung disease | Uganda | 1318 | Retrospective cohort | Not reported | Authors from country of focus and USA | Not reported | Cox Proportion hazard model | [4] |
| 5 | Olayanju et.al | 2019 | International Society for Infectious Diseases | South Africa | 63 | Prospective cohort | Not reported | Authors from country of focus and UK | Log-rank test | Cox Proportion hazard model | [5] |
| 6 | Agizew et.al | 2019 | BMC Infectious Diseases | Botswana | 6041 | Randomized Trial | Not reported | Authors from country of focus, South Africa and USA | Not reported | Cox Proportion hazard model | [6] |
| *7* | Berry et.al | 2019 | BMC Public Health | South Africa | 182890 | Retrospective cohort | Yes | Authors from country of focus and USA | Not reported | Cox Proportion hazard model | [7] |
| *8* | Huerga et.al | 2019 | BMC Infectious Diseases | Kenya | 606 | Prospective cohort | Not reported | Authors from country of focus and Europe | Log-rank test | Cox Proportion hazard model | [8] |
| *9* | Gezae et.al | 2019 | BMC Infectious Diseases | Ethiopia | 305 | Retrospective cohort | Yes | Authors from country of focus | Log-rank test | Cox Proportion hazard model | [9] |
| *10* | Woldeyohannes et.al | 2019 | PLoS ONE | Ethiopia | 415 | Retrospective cohort | Not reported | Authors from country of focus | Log-rank test | Cox Proportion hazard model | [10] |
| *11* | Bouton et.al | 2019 | Pan African Medical Journal | Ghana | 394 | Retrospective cohort | Yes | Authors from country of focus and USA | Not reported | Cox Proportion hazard model | [11] |
| *12* | Azeez et.al | 2019 | International Journal of Mycobacteriology | South Africa | 910 | Retrospective cohort | Yes | Authors from country of focus | Log-rank test | Cox Proportion hazard model | [12] |
| *13* | Kassa et.al | 2019 | BMC Infectious Diseases | Ethiopia | 332 | Retrospective cohort | Yes | Authors from country of focus | Log-rank test | Parametric method (Gompertz distribution) | [13] |
| **Number** | **Primary Author** | **Year of publication** | **Publication Journal** | **Country of study** | **N included** | **Study design** | **Statistician among authors’ affiliation** | **Type of research collaboration** | **Method of event rate comparison** | **Regression model** | **Reference** |
| *14* | Ketema et.al | 2019 | BMC Public Health | Ethiopia | 508 | Retrospective cohort | Yes | Authors from country of focus | Log-rank test | Accelerated failure time model (Weibull distribution) | [14] |
| *15* | Abdullahi et.al | 2019 | PLoS ONE | Kenya | 10717 | Retrospective cohort | Not reported | Authors from country of focus | Log-rank test | Competing risk analysis model | [15] |
| *16* | Schutz et.al | 2019 | PLoS Medicine | South Africa | 576 | Prospective cohort | Yes | Authors from country of focus, Brazil, USA, Europe and UK | Log-rank test | Cox Proportion hazard model | [16] |
| *17* | Limenih et.al | 2019 | BMC Public Health | Ethiopia | 311 | Retrospective cohort | Yes | Authors from country of focus | Wilcoxon-Breslow-Gehan test | Accelerated failure time model (Weibull distribution) | [17] |
| *18* | Bajehson et.al | 2019 | PLoS ONE | Nigeria | 147 | Retrospective cohort | Not reported | Authors from country of focus and Europe | Not reported | Cox Proportion hazard model | [18] |
| *19* | Bulabula et.al | 2019 | Clinical Infectious Diseases | Democratic Republic of Congo | 1535 | Retrospective cohort | Yes | Authors from country of focus, South Africa, Nigeria, Europe and USA | Log-rank test | Cox Proportion hazard model | [19] |
| *20* | Gupta-Wright et.al | 2019 | Clinical Infectious Diseases | Malawi and South Africa | 322 | Prospective cohort | Yes | Authors from country of focus and UK | Not reported | Cox Proportion hazard model | [20] |
| *21* | Moon et.al | 2019 | The Pediatric Infectious Disease Journal | Mozambique | 938 | Retrospective cohort | Yes | Authors from country of focus, Europe and USA | Log-rank test | Cox Proportion hazard model | [21] |
| *22* | Brust et.al | 2018 | Clinical Infectious Diseases | South Africa | 206 | Prospective cohort | Yes | Authors from country of focus and USA | Log-rank test | Cox Proportion hazard model | [22] |
| *23* | Azeez et.al | 2018 | International Journal of Mycobacteriology | South Africa | 910 | Retrospective cohort | Yes | Authors from country of focus | Log-rank test | Cox Proportion hazard model | [23] |
| *24* | Dangisso et.al | 2018 | PLoS ONE | Ethiopia | 2272 | Retrospective cohort | Not reported | Authors from country of focus, Europe and UK | Not reported | Cox Proportion hazard model | [24] |
| *25* | Atalell et.al | 2018 | PLoS ONE | Ethiopia | 271 | Retrospective cohort | Not reported | Authors from country of focus | Not reported | Cox Proportion hazard model | [25] |
| **Number** | **Primary Author** | **Year of publication** | **Publication Journal** | **Country of study** | **N included** | **Study design** | **Statistician among authors’ affiliation** | **Type of research collaboration** | **Method of event rate comparison** | **Regression model** | **Reference** |
| *26* | Onyango et.al | 2018 | The Journal of Pediatrics | Kenya | 23753 | Retrospective cohort | Not reported | Authors from country of focus, Europe and USA | Not reported | Cox Proportion hazard model | [26] |
| *27* | Hirasen et.al | 2018 | PLoS ONE | South Africa | 240 | Prospective cohort | Yes | Authors from country of focus and USA | Not reported | Cox Proportion hazard model | [27] |
| *28* | Wickett et,al | 2018 | Tropical Medicine and International Health | Liberia | 560 | Retrospective cohort | Not reported | Authors from country of focus, Europe and USA | Not reported | Competing risk analysis model | [28] |
| *29* | Bonnet et.al | 2018 | The Pediatric Infectious Disease Journal | Uganda | 360 | Prospective cohort | Not reported | Authors from country of focus and Europe | Not reported | Cox Proportion hazard model | [29] |
| *30* | Worodria et.al | 2018 | AIDS | Uganda | 387 | Prospective cohort | Yes | Authors from country of focus, USA and Europe | Not reported | Cox Proportion hazard model | [30] |
| *31* | Akalu et.al | 2018 | PLoS ONE | Ethiopia | 392 | Retrospective cohort | Yes | Authors from country of focus | Log-rank test | Parametric model (Weibull distribution) | [31] |
| *32* | Nansumba et.al | 2018 | International journal tuberculosis lung disease | Uganda | 144 | Prospective cohort | Not reported | Authors from country of focus and Europe | Not reported | Not reported^1^ | [32] |
| *33* | Schnippel et.al | 2018 | Lancet Respiratory Medicine | South Africa | 19617 | Retrospective cohort | Not reported | Authors from country of focus | Log-rank test | Cox Proportion hazard model | [33] |
| *34* | Herce et.al | 2018 | BMC Infectious Diseases | Zambia | 473 | Prospective cohort | Not reported | Authors from country of focus and USA | Log-rank test | Cox Proportion hazard model | [34] |
| *35* | Verdecchia et.al | 2018 | PLoS ONE | Swaziland (eSwatini) | 174 | Retrospective cohort | Not reported | Authors from country of focus, Europe and UK | Not reported | Cox Proportion hazard model | [35] |
| *36* | Kaplan et.al | 2018 | BMC Infectious Diseases | South Africa | 118989 | Retrospective cohort | Not reported | Authors from country of focus, Uganda and Europe | Log-rank test | Cox Proportion hazard model | [36] |
| *37* | Shibabaw et.al | 2018 | PLoS ONE | Ethiopia | 235 | Retrospective cohort | Not reported | Authors from country of focus and USA | Log-rank test | Cox Proportion hazard model | [37] |
| *38* | Adamu et.al | 2017 | PLoS ONE | Nigeria | 299 | Retrospective cohort | Not reported | Authors from country of focus, USA and UK | Not reported | Cox Proportion hazard model | [38] |
| **Number** | **Primary Author** | **Year of publication** | **Publication Journal** | **Country of study** | **N included** | **Study design** | **Statistician among authors’ affiliation** | **Type of research collaboration** | **Method of event rate comparison** | **Regression model** | **Reference** |
| *39* | Alene et.al | 2017 | Tropical Medicine and International Health | Ethiopia | 242 | Retrospective cohort | Yes | Authors from country of focus and Australia | Log-rank test | Cox Proportion hazard model | [39] |
| *40* | Adamu et.al | 2017 | BMC Infectious Diseases | Nigeria | 1424 | Retrospective cohort | Not reported | Authors from country of focus and UK | Log-rank test | Cox Proportion hazard model | [40] |
| *41* | Schnippel et.al | 2017 | International journal tuberculosis lung disease | South Africa | 20653 | Retrospective cohort | Yes | Authors from country of focus and USA | Log-rank test | Cox Proportion hazard model | [41] |
| *42* | Hall et.al | 2017 | The Pediatric Infectious Disease Journal | South Africa | 423 | Retrospective cohort | Yes | Authors from country of focus and USA | Log-rank test | Cox Proportion hazard model | [42] |
| *43* | Onyango et.al | 2017 | PLoS ONE | Kenya | 162014 | Retrospective cohort | Not reported | Authors from country of focus and USA | Log-rank test | Cox Proportion hazard model | [43] |
| *44* | Kapata et.al | 2017 | Infection | Zambia | 110 | Retrospective cohort | Yes | Authors from country of focus, Europe and UK | Log-rank test | Cox Proportion hazard model | [44] |
| *45* | Mohr et.al | 2017 | PLoS ONE | South Africa | 404 | Prospective cohort | Yes | Authors from country of focus | Not reported | Not reported^1^ | [45] |
| *46* | Beyene et.al | 2016 | BMC Public Health | Ethiopia | 1260 | Retrospective cohort | Not reported | Authors from country of focus | Log-rank test | Cox Proportion hazard model | [46] |
| *47* | Olaleye et.al | 2016 | Infectious Diseases | South Africa | 442 | Retrospective cohort | Not reported | Authors from country of focus | Log-rank test | Cox Proportion hazard model | [47] |
| *48* | Workneh et.al | 2016 | Infectious Diseases of Poverty | Ethiopia | 1314 | Prospective cohort | Not reported | Authors from country of focus and Europe | Log-rank test | Cox Proportion hazard model | [48] |
| *49* | Masini et.al | 2016 | PLoS ONE | Kenya | 90170 | Retrospective cohort | Not reported | Authors from country of focus and USA | Log-rank test | Cox Proportion hazard model | [49] |
| *50* | Gesesew et.al | 2016 | Infectious Diseases of Poverty | Ethiopia | 272 | Retrospective cohort | Yes | Authors from country of focus, Australia and Canada | Not reported | Cox Proportion hazard model | [50] |
| *51* | Kaplan et.al | 2016 | International journal tuberculosis lung disease | South Africa | 11896 | Retrospective cohort | Not reported | Authors from country of focus, Uganda and Europe | Not reported | Cox Proportion hazard model | [51] |
| **Number** | **Primary Author** | **Year of publication** | **Publication Journal** | **Country of study** | **N included** | **Study design** | **Statistician among authors’ affiliation** | **Type of research collaboration** | **Method of event rate comparison** | **Regression model** | **Reference** |
| *52* | Birlie et.al | 2015 | PLoS ONE | Ethiopia | 810 | Retrospective cohort | Yes | Authors from country of focus | Log-rank test | Cox Proportion hazard model | [52] |
| *53* | Moyo et.al | 2015 | PLoS ONE | South Africa | 452 | Retrospective cohort | Yes | Authors from country of focus | Log-rank test | Cox Proportion hazard model | [53] |
| *54* | Meressa et.al | 2015 | BMJ Thorax | Ethiopia | 612 | Retrospective cohort | Not reported | Authors from country of focus, Cambodia and USA | Log-rank test | Cox Proportion hazard model | [54] |
| *55* | Daniels et.al | 2015 | PLoS ONE | South Africa | 982 | Retrospective cohort | Yes | Authors from country of focus and Europe | Not reported | Cox Proportion hazard model | [55] |
| *56* | Wejse et.al | 2015 | International Journal of Infectious Diseases | Guinea-Bissau | 1312 | Prospective | Not reported | Authors from country of focus and Europe | Not reported | Cox Proportion hazard model | [56] |
| *57* | Acuna-Villaorduna et.al | 2015 | America Journal of Tropical Medicine and hygiene | Uganda | 284 | Retrospective cohort | Yes | Authors from country of focus and USA | Log-rank test | Cox Proportion hazard model | [57] |
| *58* | Pepper et.al | 2015 | AIDS Research and Therapy | South Africa | 16209 | Retrospective cohort | Yes | Authors from country of focus, UK and USA | Not reported | Cox Proportion hazard model | [58] |
| *59* | Cox et.al | 2014 | International journal tuberculosis lung disease | South Africa | 1208 | Retrospective cohort | Yes | Authors from country of focus | Not reported | Cox Proportion hazard model | [59] |
| *60* | Kirenga et.al | 2014 | PLoS ONE | Uganda | 96 | Prospective Cohort | Yes | Authors from country of focus, South Africa, UK and USA | Not reported | Cox Proportion hazard model | [60] |
| *61* | Bekker et.al | 2014 | International journal tuberculosis lung disease | South Africa | 56 | Prospective Cohort | Not reported | Authors from country of focus | Not reported | Cox Proportion hazard model | [61] |
| *62* | Zetola et.al | 2014 | The Journal of Infectious Diseases | Botswana | 475 | Retrospective cohort | Not reported | Authors from country of focus and USA | Not reported | Cox Proportion hazard model | [62] |
| *63* | Getachew et.al | 2013 | International journal of Pharmaceutical Sciences and Research | Ethiopia | 188 | Retrospective cohort | Not reported | Authors from country of focus | Log-rank test | Cox Proportion hazard model | [63] |
| *64* | Sileshi et.al | 2013 | BMC Infectious Diseases | Ethiopia | 422 | Retrospective cohort | Yes | Authors from country of focus and USA | Log-rank test | Cox Proportion hazard model | [64] |
| **Number** | **Primary Author** | **Year of publication** | **Publication Journal** | **Country of study** | **N included** | **Study design** | **Statistician among authors’ affiliation** | **Type of research collaboration** | **Method of event rate comparison** | **Regression model** | **Reference** |
| *65* | Hafkin et.al | 2013 | International journal tuberculosis lung disease | Botswana | 70 | Prospective Cohort | Yes | Authors from country of focus and USA | Log-rank test | Cox Proportion hazard model | [65] |
| *66* | Kendall et.al | 2013 | PLoS ONE | South Africa | 225 | Retrospective cohort | Yes | Authors from country of focus and USA | Not reported | Cox Proportion hazard model | [66] |
| *67* | Henegar et.al | 2012 | International journal tuberculosis lung disease | Democratic Republic of Congo | 5685 | Retrospective cohort | Yes | Authors from country of focus and USA | Not reported | Cox Proportion hazard model | [67] |
| *68* | Marx et.al | 2012 | PLoS ONE | South Africa | 2136 | Retrospective cohort | Not reported | Authors from country of focus and Europe | Not reported | Cox Proportion hazard model | [68] |
| *69* | Visser et.al | 2012 | PLoS ONE | South Africa | 113 | Prospective cohort | Yes | Authors from country of focus and Europe | Log-rank test | Cox Proportion hazard model | [69] |
| *70* | Mupere et.al | 2012 | Annals of Epidemiology | Uganda | 747 | Retrospective cohort | Yes | Authors from country of focus and USA | Log-rank test | Cox Proportion hazard model | [70] |
| *71* | Farley et.al | 2011 | PLoS ONE | South Africa | 757 | Prospective cohort | Yes | Authors from country of focus and USA | Log-rank test | Competing risk analysis model | [71] |
| *72* | Getahun et.al | 2011 | BMC Infectious Diseases | Ethiopia | 6450 | Retrospective cohort | Yes | Authors from country of focus | Log-rank test | Cox Proportion hazard model | [72] |
| *73* | Worodria et.al | 2011 | Journal of Acquired Immune Deficiency Syndromes | Uganda | 302 | Prospective cohort | Yes | Authors from country of focus and Europe | Log-rank test | Cox Proportion hazard model | [73] |
| *74* | Jones-Lopez et.al | 2011 | PLoS Medicine | Uganda | 288 | Prospective cohort | Yes | Authors from country of focus, South Africa, USA and UK | Not reported | Cox Proportion hazard model | [74] |
| *75* | Dheda et.al | 2010 | Lancet | South Africa | 195 | Retrospective cohort | Yes | Authors from country of focus, USA and UK | Log-rank test | Cox Proportion hazard model | [75] |
| *76* | Datiko et.al | 2010 | International journal tuberculosis lung disease | Ethiopia | 725 | Retrospective cohort | Not reported | Authors from country of focus and Europe | Log-rank test | Cox Proportion hazard model | [76] |
| ^1^ The two studies reported only survival curves. | | | | | | | | | | | |

# **References**

1. Tola HH, Holakouie-Naieni K, Mansournia MA, Yaseri M, Tesfaye E, Mahamed Z, et al. Low enrollment and high treatment success in children with drug-resistant tuberculosis in Ethiopia: A ten years national retrospective cohort study. PLoS One. 2020.

2. Shaweno T, Getnet M, Fikru C. Does time to loss to follow-up differ among adult tuberculosis patients initiated on tuberculosis treatment and care between general hospital and health centers? A retrospective cohort study. Trop Med Health. 2020.

3. Schwœbel V, Trébucq A, Kashongwe Z, Bakayoko AS, Kuaban C, Noeske J, et al. Outcomes of a nine-month regimen for rifampicin-resistant tuberculosis up to 24 months after treatment completion in nine African countries. EClinicalMedicine. 2020.

4. Musaazi J, Sekaggya-Wiltshire C, Kiragga AN, Kalule I, Reynolds SJ, Manabe YC, et al. Sustained positive impact on tuberculosis treatment outcomes of TB-HIV integrated care in Uganda. Int J Tuberc Lung Dis. 2019.

5. Olayanju O, Esmail A, Limberis J, Gina P, Dheda K. Linezolid interruption in patients with fluoroquinolone-resistant tuberculosis receiving a bedaquiline-based treatment regimen. Int J Infect Dis. 2019.

6. Agizew T, Chihota V, Nyirenda S, Tedla Z, Auld AF, Mathebula U, et al. Tuberculosis treatment outcomes among people living with HIV diagnosed using Xpert MTB/RIF versus sputum-smear microscopy in Botswana: A stepped-wedge cluster randomised trial. BMC Infect Dis. 2019.

7. Berry KM, Rodriguez CA, Berhanu RH, Ismail N, Mvusi L, Long L, et al. Treatment outcomes among children, adolescents, and adults on treatment for tuberculosis in two metropolitan municipalities in Gauteng Province, South Africa. BMC Public Health. 2019.

8. Huerga H, Ferlazzo G, Wanjala S, Bastard M, Bevilacqua P, Ardizzoni E, et al. Mortality in the first six months among HIV-positive and HIV-negative patients empirically treated for tuberculosis. BMC Infect Dis. 2019.

9. Gezae KE, Abebe HT, Gebretsadik LG. Incidence and predictors of LTFU among adults with TB/HIV co-infection in two governmental hospitals, Mekelle, Ethiopia, 2009-2016: Survival model approach. BMC Infect Dis. 2019.

10. Woldeyohannes D, Assefa T, Aman R, Tekalegn Y, Hailemariam Z. Predictors of time to unfavorable treatment outcomes among patients with multidrug resistant tuberculosis in Oromia region, Ethiopia. PLoS One. 2019.

11. Bouton TC, Forson A, Kudzawu S, Zigah F, Jenkins H, Bamfo TD, et al. High mortality during tuberculosis retreatment at a ghanaian tertiary center: A retrospective cohort study. Pan Afr Med J. 2019.

12. Azeez A, Mutambayi R, Odeyemi A, Ndege J. Survival model analysis of tuberculosis treatment among patients with human immunodeficiency virus coinfection. Int J Mycobacteriology. 2019.

13. Kassa GM, Teferra AS, Wolde HF, Muluneh AG, Merid MW. Incidence and predictors of lost to follow-up among drug-resistant tuberculosis patients at University of Gondar Comprehensive Specialized Hospital, Northwest Ethiopia: A retrospective follow-up study. BMC Infect Dis. 2019.

14. Ketema DB, Muchie KF, Andargie AA. Time to poor treatment outcome and its predictors among drug-resistant tuberculosis patients on second-line anti-Tuberculosis treatment in Amhara region, Ethiopia: Retrospective cohort study. BMC Public Health. 2019.

15. Abdullahi OA, Ngari MM, Sanga D, Katana G, Willetts A. Mortality during treatment for tuberculosis; a review of surveillance data in a rural county in Kenya. PLoS One. 2019.

16. Schutz C, Barr D, Andrade BB, Shey M, Ward A, Janssen S, et al. Clinical, microbiologic, and immunologic determinants of mortality in hospitalized patients with HIV-associated tuberculosis: A prospective cohort study. PLoS Med. 2019.

17. Limenih YA, Workie DL. Survival analysis of time to cure on multi-drug resistance tuberculosis patients in Amhara region, Ethiopia. BMC Public Health. 2019.

18. Bajehson M, Musa BM, Gidado M, Nsa B, Sani U, Habibu AT, et al. Determinants of mortality among patients with drug-resistant tuberculosis in northern Nigeria. PLoS One. 2019.

19. Bulabula ANH, Nelson JA, Musafiri EM, MacHekano R, Sam-Agudu NA, Diacon AH, et al. Prevalence, Predictors, and Successful Treatment Outcomes of Xpert MTB/RIF-identified Rifampicin-resistant Tuberculosis in Post-conflict Eastern Democratic Republic of the Congo, 2012-2017: A Retrospective Province-Wide Cohort Study. Clin Infect Dis. 2019.

20. Gupta-Wright A, Fielding K, Wilson D, van Oosterhout JJ, Grint D, Mwandumba HC, et al. Tuberculosis in Hospitalized Patients With Human Immunodeficiency Virus: Clinical Characteristics, Mortality, and Implications From the Rapid Urine-based Screening for Tuberculosis to Reduce AIDS Related Mortality in Hospitalized Patients in Africa. Clin Infect Dis. 2019.

21. Moon TD, Nacarapa E, Verdu ME, MacUácua S, Mugabe D, Gong W, et al. Tuberculosis Treatment Outcomes among Children in Rural Southern Mozambique: A 12-year Retrospective Study. Pediatr Infect Dis J. 2019.

22. Brust JCM, Shah NS, Mlisana K, Moodley P, Allana S, Campbell A, et al. Improved Survival and Cure Rates with Concurrent Treatment for Multidrug-Resistant Tuberculosis-Human Immunodeficiency Virus Coinfection in South Africa. Clin Infect Dis. 2018.

23. Azeez A, Ndege J, Mutambayi R. Associated factors with unsuccessful tuberculosis treatment outcomes among tuberculosis/HIV coinfected patients with drug-resistant tuberculosis. Int J Mycobacteriology. 2018.

24. Dangisso MH, Woldesemayat EM, Datiko DG, Lindtjørn B. Long-term outcome of smear-positive tuberculosis patients after initiation and completion of treatment: A ten-year retrospective cohort study. PLoS One. 2018.

25. Atalell KA, Tebeje NB, Ekubagewargies DT. Survival and predictors of mortality among children co-infected with tuberculosis and human immunodeficiency virus at University of Gondar Comprehensive Specialized Hospital, Northwest Ethiopia. A retrospective follow-up study. PLoS One. 2018.

26. Onyango DO, Yuen CM, Masini E, Borgdorff MW. Epidemiology of Pediatric Tuberculosis in Kenya and Risk Factors for Mortality during Treatment: A National Retrospective Cohort Study. J Pediatr. 2018.

27. Hirasen K, Berhanu R, Evans D, Rosen S, Sanne I, Long L. High rates of death and loss to follow-up by 12 months of rifampicin resistant TB treatment in South Africa. PLoS One. 2018.

28. Wickett E, Peralta-Santos A, Beste J, Micikas M, Toe F, Rogers J, et al. Treatment outcomes of TB-infected individuals attending public sector primary care clinics in rural Liberia from 2015 to 2017: a retrospective cohort study. Trop Med Int Heal. 2018.

29. Bonnet M, Nansumba M, Bastard M, Orikiriza P, Kyomugasho N, Nansera D, et al. Outcome of children with presumptive tuberculosis in Mbarara, Rural Uganda. Pediatr Infect Dis J. 2018.

30. Worodria W, Ssempijja V, Hanrahan C, Ssegonja R, Muhofwa A, Mazapkwe D, et al. Opportunistic diseases diminish the clinical benefit of immediate co-infected antiretroviral adults with therapy low CD4 in HIV-tuberculosis R cell counts. AIDS. 2018.

31. Yihunie Akalu T, Fentahun Muchie K, Alemu Gelaye K. Time to sputum culture conversion and its determinants among Multi-drug resistant Tuberculosis patients at public hospitals of the Amhara Regional State: A multicenter retrospective follow up study. PLoS One. 2018.

32. Nansumba M, Kumbakumba E, Orikiriza P, Bastard M, Mwanga JA, Boum Y, et al. Treatment outcomes and tolerability of the revised WHO anti-tuberculosis drug dosages for children. Int J Tuberc Lung Dis. 2018.

33. Schnippel K, Ndjeka N, Maartens G, Meintjes G, Master I, Ismail N, et al. Effect of bedaquiline on mortality in South African patients with drug-resistant tuberculosis: a retrospective cohort study. Lancet Respir Med. 2018.

34. Herce ME, Morse J, Luhanga D, Harris J, Smith HJ, Besa S, et al. Integrating HIV care and treatment into tuberculosis clinics in Lusaka, Zambia: Results from a before-after quasi-experimental study. BMC Infect Dis. 2018.

35. Verdecchia M, Keus K, Blankley S, Vambe D, Ssonko C, Piening T, et al. Model of care and risk factors for poor outcomes in patients on multi-drug resistant tuberculosis treatment at two facilities in eSwatini (formerly Swaziland), 2011-2013. PLoS One. 2018.

36. Kaplan R, Hermans S, Caldwell J, Jennings K, Bekker LG, Wood R. HIV and TB co-infection in the ART era: CD4 count distributions and TB case fatality in Cape Town. BMC Infect Dis. 2018.

37. Shibabaw A, Gelaw B, Wang SH, Tessema B. Time to sputum smear and culture conversions in multidrug resistant tuberculosis at university of gondar hospital, northwest Ethiopia. PLoS One. 2018.

38. Adamu AL, Aliyu MH, Galadanci NA, Musa BM, Gadanya MA, Gajida AU, et al. Deaths during tuberculosis treatment among paediatric patients in a large tertiary hospital in Nigeria. PLoS One. 2017.

39. Alene KA, Viney K, McBryde ES, Tsegaye AT, Clements ACA. Treatment outcomes in patients with multidrug-resistant tuberculosis in north-west Ethiopia. Trop Med Int Heal. 2017.

40. Adamu AL, Gadanya MA, Abubakar IS, Jibo AM, Bello MM, Gajida AU, et al. High mortality among tuberculosis patients on treatment in Nigeria: A retrospective cohort study. BMC Infect Dis. 2017.

41. Schnippel K, Firnhaber C, Ndjeka N, Conradie F, Page-Shipp L, Berhanu R, et al. Persistently high early mortality despite rapid diagnostics for drug-resistant tuberculosis cases in South Africa. Int J Tuberc Lung Dis. 2017.

42. Hall EW, Morris SB, Moore BK, Erasmus L, Odendaal R, Menzies H, et al. Treatment Outcomes of Children with HIV Infection and Drug-Resistant TB in Three Provinces in South Africa, 2005-2008. Pediatr Infect Dis J. 2017.

43. Onyango DO, Yuen CM, Cain KP, Ngari F, Masini EO, Borgdorff MW. Reduction of HIV-associated excess mortality by antiretroviral treatment among tuberculosis patients in Kenya. PLoS One. 2017.

44. Kapata N, Grobusch MP, Chongwe G, Chanda-Kapata P, Ngosa W, Tembo M, et al. Outcomes of multidrug-resistant tuberculosis in Zambia: a cohort analysis. Infection. 2017.

45. Mohr E, Daniels J, Beko B, Isaakidis P, Cox V, Steele SJ, et al. DOT or SAT for Rifampicin-resistant tuberculosis? A non-randomized comparison in a high HIV-prevalence setting. PLoS One. 2017.

46. Beyene Y, Geresu B, Mulu A. Mortality among tuberculosis patients under DOTS programme: A historical cohort study. BMC Public Health. 2016.

47. Olaleye AO, Beke AK. Survival of smear-positive multidrug resistant tuberculosis patients in Witbank, South Africa: A retrospective cohort study. Infect Dis (Auckl). 2016.

48. Workneh MH, Bjune GA, Yimer SA. Diabetes mellitus is associated with increased mortality during tuberculosis treatment: A prospective cohort study among tuberculosis patients in South-Eastern Amahra Region, Ethiopia. Infect Dis Poverty. 2016.

49. Masini EO, Mansour O, Speer CE, Addona V, Hanson CL, Sitienei JK, et al. Using survival analysis to identify risk factors for treatment interruption among new and retreatment tuberculosis patients in Kenya. PLoS One. 2016.

50. Gesesew H, Tsehayneh B, Massa D, Gebremedhin A, Kahsay H, Mwanri L. Predictors of mortality in a cohort of tuberculosis/HIV co-infected patients in Southwest Ethiopia. Infect Dis Poverty. 2016.

51. Kaplan R, Caldwell J, Hermans S, Adriaanse S, Mtwisha L, Bekker LG, et al. An integrated community TB-HIV adherence model provides an alternative to DOT for tuberculosis patients in Cape Town. Int J Tuberc Lung Dis. 2016.

52. Birlie A, Tesfaw G, Dejene T, Woldemichael K. Time to death and associated factors among tuberculosis patients in dangila woreda, northwest Ethiopia. PLoS One. 2015.

53. Moyo S, Cox HS, Hughes J, Daniels J, Synman L, De Azevedo V, et al. Loss from treatment for drug resistant tuberculosis: Risk factors and patient outcomes in a community-based program in khayelitsha, South Africa. PLoS One. 2015.

54. Meressa D, Hurtado RM, Andrews JR, Diro E, Abato K, Daniel T, et al. Achieving high treatment success for multidrug-resistant TB in Africa: Initiation and scale-up of MDR TB care in Ethiopia - An observational cohort study. Thorax. 2015.

55. Daniels JF, Khogali M, Mohr E, Cox V, Moyo S, Edginton M, et al. Time to ART initiation among patients treated for rifampicin-resistant tuberculosis in khayelitsha, South Africa: Impact on mortality and treatment success. PLoS One. 2015.

56. Wejse C, Patsche CB, Kühle A, Bamba FJV, Mendes MS, Lemvik G, et al. Impact of HIV-1, HIV-2, and HIV-1+2 dual infection on the outcome of tuberculosis. Int J Infect Dis. 2015.

57. Acuña-Villaorduña C, Ayakaka I, Dryden-Peterson S, Nakubulwa S, Worodria W, Reilly N, et al. High mortality associated with retreatment of tuberculosis in a clinic in Kampala, Uganda: A retrospective study. Am J Trop Med Hyg. 2015.

58. Pepper DJ, Schomaker M, Wilkinson RJ, Azevedo V, Maartens G. Independent predictors of tuberculosis mortality in a high HIV prevalence setting: A retrospective cohort study. AIDS Res Ther. 2015.

59. Cox H, Hughes J, Daniels J, Azevedo V, McDermid C, Poolman M, et al. Community-based treatment of drug-resistant tuberculosis in Khayelitsha, South Africa. Int J Tuberc Lung Dis. 2014.

60. Kirenga BJ, Levin J, Ayakaka I, Worodria W, Reilly N, Mumbowa F, et al. Treatment outcomes of new tuberculosis patients hospitalized in Kampala, Uganda: A prospective cohort study. PLoS One. 2014.

61. Bekker A, Slogrove AL, Schaaf HS, Du Preez K, AHesseling C. Determinants of tuberculosis treatment completion among newborns in a high-burden setting. Int J Tuberc Lung Dis. 2014.

62. Zetola NM, Modongo C, Moonan PK, Ncube R, Matlhagela K, Sepako E, et al. Clinical outcomes among persons with pulmonary tuberculosis caused by Mycobacterium tuberculosis isolates with phenotypic heterogeneity in results of drug-susceptibility tests. J Infect Dis. 2014.

63. Getachew T, Bayray A, Weldearegay B. Survival and predictors of mortality among patients under multi-drug resistant tuberculosis treatment in Ethiopia: St. Peter’s specialized tuberculosis hospital, Ethiopia. Int J Pharm Sci Res. 2013.

64. Sileshi B, Deyessa N, Girma B, Melese M, Suarez P. Predictors of mortality among TB-HIV Co-infected patients being treated for tuberculosis in Northwest Ethiopia: A retrospective cohort study. BMC Infect Dis. 2013.

65. Hafkin J, Modongo C, Newcomb C, Lowenthal E, MacGregor RR, Steenhoff AP, et al. Impact of the human immunodeficiency virus on early multidrug-resistant tuberculosis treatment outcomes in Botswana. Int J Tuberc Lung Dis. 2013.

66. Kendall EA, Theron D, Franke MF, Van Helden P, Victor TC, Murray MB, et al. Alcohol, hospital discharge, and socioeconomic risk factors for default from multidrug resistant tuberculosis treatment in rural South Africa: A retrospective cohort study. PLoS One. 2013.

67. Henegar CE, Behets F, Vanden Driessche K, Tabala M, Bahati E, Bola V, et al. Mortality among tuberculosis patients in the Democratic Republic of Congo. Int J Tuberc Lung Dis. 2012.

68. Marx FM, Dunbar R, Enarson DA, Beyers N. The Rate of Sputum Smear-Positive Tuberculosis after Treatment Default in a High-Burden Setting: A Retrospective Cohort Study. PLoS One. 2012.

69. Visser ME, Stead MC, Walzl G, Warren R, Schomaker M, Grewal HMS, et al. Baseline predictors of sputum culture conversion in pulmonary tuberculosis: Importance of cavities, smoking, time to detection and w-beijing genotype. PLoS One. 2012.

70. Mupere E, Malone L, Zalwango S, Chiunda A, Okwera A, Parraga I, et al. Lean Tissue Mass Wasting is Associated With Increased Risk of Mortality Among Women With Pulmonary Tuberculosis in Urban Uganda. Ann Epidemiol. 2012.

71. Farley JE, Ram M, Pan W, Waldman S, Cassell GH, Chaisson RE, et al. Outcomes of multi-drug resistant tuberculosis (MDR-TB) among a cohort of South African patients with high HIV prevalence. PLoS One. 2011.

72. Getahun B, Ameni G, Biadgilign S, Medhin G. Mortality and associated risk factors in a cohort of tuberculosis patients treated under DOTS programme in Addis Ababa, Ethiopia. BMC Infect Dis. 2011.

73. Worodria W, Massinga-Loembe M, Mazakpwe D, Luzinda K, Menten J, Van Leth F, et al. Incidence and predictors of mortality and the effect of tuberculosis immune reconstitution inflammatory syndrome in a cohort of TB/HIV patients commencing antiretroviral therapy. J Acquir Immune Defic Syndr. 2011.

74. Jones-López EC, Ayakaka I, Levin J, Reilly N, Mumbowa F, Dryden-Peterson S, et al. Effectiveness of the standard WHO recommended retreatment regimen (Category II) for tuberculosis in Kampala, Uganda: A prospective cohort study. PLoS Med. 2011.

75. Dheda K, Shean K, Zumla A, Badri M, Streicher EM, Page-Shipp L, et al. Early treatment outcomes and HIV status of patients with extensively drug-resistant tuberculosis in South Africa: a retrospective cohort study. Lancet. 2010.

76. Datiko DG, Lindtjørn B. Mortality in successfully treated tuberculosis patients in southern Ethiopia: Retrospective follow-up study. Int J Tuberc Lung Dis. 2010.
